# Surgical ablation for atrial fibrillation: impact of Diabetes Mellitus type 2

**DOI:** 10.1186/s12933-023-01810-x

**Published:** 2023-03-31

**Authors:** Alexander Kogan, Avishay Grupper, Avi Sabbag, Eilon Ram, Tamer Jamal, Eyal Nof, Enrique Z. Fisman, Shany Levin, Roy Beinart, Jonathan Frogel, Ehud Raanani, Leonid Sternik

**Affiliations:** 1 Department of Cardiac Surgery, Sheba Medical Center at Tel Hashomer, 52621 Ramat Gan, Israel; 2 Division of Cardiology, Sheba Medical Center at Tel Hashomer, 52621 Ramat Gan, Israel; 3 Department of Anesthesiology, Sheba Medical Center at Tel Hashomer, 52621 Ramat Gan, Israel; 4 Sackler School of Medicine, Tel Aviv University, Tel Aviv, Israel

**Keywords:** Atrial fibrillation, Diabetes mellitus type 2, Surgical ablation, Long-term follow-up

## Abstract

**Background:**

Diabetes mellitus (DM) type 2 is an independent risk factor for atrial fibrillation (AF). Surgical ablation or "maze procedure" is an option for patients with AF undergoing concomitant or isolated cardiac surgery. The aim of this study was to evaluate the impact of DM type 2 on early and long-term outcomes of patients following surgical AF ablation.

**Methods:**

We performed an observational cohort study in Israel’s largest tertiary care center. All data of patients who underwent surgical AF ablation, between 2006 and 2021 were extracted from our departmental database. Patients were divided into Group I (non-diabetic patients) and Group II (DM type 2 patients). We compared the two groups with respect to freedom from recurrent atrial arrhythmia, and mortality rate.

**Results:**

The study population included 606 patients. Group I (non-DM patients), consisting of 484 patients, and Group II (DM type 2 patients), comprised 122 patients. Patients with DM were older, had more hypertension and incidence of cerebrovascular accident (CVA)/transient ischemic attack (TIA), higher EuroSCORE (*p* < .05 for all), and a longer bypass time—130 ± 40 vs. 122 ± 36 min (*p* = 0.028). The mean follow-up duration was 39.0 ± 22.7 months. Freedom from atrial fibrillation was similar between the non-DM and DM type 2 groups after a 1-year follow-up, 414 (88.2%) vs. 101 (87.1%) (*p* = 0.511), after a 3-year follow-up, 360 (86.3%) vs. 84 (79.9%) (*p* = 0.290) and after a 5-year follow-up, 226 (74.1%) vs. 55 (71.5%) (*p* = 0.622) respectively. Furthermore, 1- and 3-year mortality was similar between non-DM and DM type 2 groups, 2.5% vs. 4.9%, (*p* = 0.226) and 5.6% vs. 10.5% (*p* = 0.076) respectively. 5-year mortality was higher in Group II (DM type 2 patients) compared with Group I (non-DM patients), 11.1% vs. 23.4% (*p* = 0.009).

**Conclusion:**

Surgical ablation had a high success rate, with freedom from recurrent atrial arrhythmia at 1- 3- and 5- years follow-up in both the DM type 2 and non-DM groups. Furthermore,1- and 3-year mortality after surgical ablation was also similar in both groups. However, 5-year mortality was higher in the DM type 2 group.

## Introduction

Diabetes mellitus type 2 (DM) is a risk factor for the development of atrial fibrillation (AF) [1]. Prevalence varied between 5.3% in diabetic population older than 18 years [2] to 25% in the population older than 65 years [3]. In addition, the incidence of AF may depend on the received anti-glibetic treatment. It has been shown that DM type 2 patients treated with sodium-glucose co-transporter inhibitors (SGLT2i) present a lower incidence of AF [4]. The presence of DM type 2 in patients with AF is associated with an increased risk of cardiovascular and all-cause mortality [5]. Surgical AF ablation (or “maze procedure’’) is approved in the current guidelines for patients with AF undergoing concomitant cardiac surgery [6]. Concomitant AF surgery approximately doubles the likelihood of freedom from AF, atrial flutter, or atrial tachycardia with a small absolute increase in needing a permanent pacemaker [7]. Surgical ablation confers significant rhythm and survival benefits without additional operative risk [8]. On the other hand, there are studies [9] that show that DM is associated with an increased risk of major adverse events. However, data regarding the efficiency and safety of concomitant cardiac surgery and surgical AF ablation in diabetic patients are absent.

The aim of this study was to compare short- and long-term outcomes and procedural failure in DM type 2 and non-DM patients who underwent the surgical ablation of AF. Outcomes variables include cardiac rhythm at 1- 3- and 5-year follow-up and 1- 3- and 5-year mortality.

## Methods

### Ethical statement

The study was approved by the Sheba Medical Center Institutional Ethics Committee (Protocol No 4257). The requirement for informed consent was waived because of the study’s retrospective nature. We carried out a retrospective, observational study of prospectively collected data from consecutive patients who underwent surgical ablation of AF with or without concomitant procedures.

AF can be divided into paroxysmal, persistent, long-standing, and permanent based on duration and ability to cardiovert [6].

Patients were divided into two groups: Group I (non-DM patients), and Group II (patients suffering from DM type 2). There were no patients with type 1 diabetes mellitus in our cohort. DM type 2 was defined in accordance with the American Diabetes Association as (a) hemoglobin A1C ≥ 6.5%; (b) fasting plasma glucose levels ≥ 126 mg/dL (7 mmol/L); (c) classic symptoms of hyperglycemia or a hyperglycemic crisis, a random plasma glucose level ≥ 200 mg/dL (11.1 mmol/L) [10]; or (d) currently on pharmacologic treatment (oral antihyperglycemic drugs and/or insulin).

Using de-identified patient data from our department's database, we evaluated the following variables: gender, age, chronic obstructive pulmonary disease, New York Heart Association heart failure functional class, DM type 2, EuroSCORE I (calculated by the surgeon) [11], dialysis-dependent renal failure, peripheral vascular disease, previous cardiovascular history, CVA/TIA, systemic and pulmonary hypertension, left ventricular function, and previous cardiac surgery. Perioperative variables studied included perioperative acute kidney injury, duration of intra-operative cross-clamp time, and cardiopulmonary bypass time.

### Surgical technique

Two surgeons (L.S. and E.R.) performed the ablation procedure for all patients. All patients underwent an ablation procedure through a mid sternotomy incision under cardioplegic arrest. The following procedures were performed: (a) right and left atrial ablation; (b) isolated left atrial ablation. The following ablation devices were used: bipolar RF device (Cardioblate 2; Medtronic Inc, Minneapolis, Minn), with the addition of a cryoprobe (Frigitronics; Cooper Surgical, Trumbull, Conn). Atrial and ventricular pacing wires were placed in all patients at the surgery.

After surgery, all patients were admitted to the intensive care unit (ICU). In order to suppress ectopic atrial activity, atrial pacing was maintained whenever possible in the early post-operative period. Patients did not receive pharmacologic prophylactic therapy of any kind, although amiodarone was administered in cases of postoperative atrial fibrillation. Oral anticoagulation (warfarin) treatment was initiated on the first postoperative day and was continued for a minimum of 3 months. Continuous electrocardiographic monitoring (telemetry) was performed on all patients throughout hospitalization. In patients with recurrent atrial fibrillation of more than 48 h, cardioversion was attempted prior to hospital discharge and, when necessary at 3 months follow-up. All patients were seen by a surgeon 1 month after discharge and then by an electrophysiologist at 3, 6, and 12 months after surgery and every 6 months thereafter. Twenty-four–hour Holter electrocardiographic monitoring was performed in all patients at either 3 or 6 months after the operation and then at 1 year after surgery and thereafter as needed but at least once a year. If a patient reported symptoms, Holter monitoring was performed immediately. All medical data from visits to any medical facilities concerning the patients’ heart rhythms were also recorded. Echocardiography was performed 6 months after surgery. Any adverse cardiovascular events were recorded. Atrial tachyarrhythmia (ATa) recurrence was defined as the detection of AF, atrial flutter, or atrial tachycardia (≥ 30 s) after a 3-month blanking period.

In the operating room and the ICU, patients from both groups received intravenous continuous infusion of regular insulin according to the Society of Thoracic Surgeons practice guideline series [12]. After discharge from the ICU, patients from Group I (non-diabetic) did not receive insulin or any other hypoglycemic medication. Patients from Group II were restarted on their preoperative anti-glycemic regimen (per oral drugs and/or insulin) as soon as they resumed regular eating.

### Database management and statistical analysis

All patient data were reviewed, corrected, and entered into a database at discharge. Descriptive statistics were used to summarize data, and numerical data were expressed as means (SD). The normality of continuous data variables distribution was analyzed using the Kolmogorov—Smirnov test. Since not all numerical data were distributed normally, Mann—Whitney U-tests were used to evaluate differences between groups. Differences between the frequencies of categorical variables were also estimated using Fisher’s exact test. All outcome variables were compared between whole groups of patients.

A Cox proportional hazard model was constructed to assess the association between DM, cardiac rhythm, and mortality. To address the differences in baseline characteristics between the groups, the subjects’ propensity score is first estimated, and then the outcome is regressed on the estimated propensity score. The regression choice depends on the nature of the outcome, therefore we used logistic regression for the predicted probability that specifies the propensity score. Variables that were associated with cardiac rhythm and mortality, adjusted to age were included in the regression model. In addition, we included pre-specified clinically significant variables in the model. The variables included in the final model were: gender, age, DM, and previous cardiac surgery. To address multiplicity we adjust the significant level by dividing by 5 (α = 0.01). In addition, Kaplan—Meier analysis was performed on cardiac rhythm among the groups, with statistical differences tested using the log-rank test. Statistical significance was assumed when the null hypothesis could be rejected at P < 0.05. All P-values are the results of two-sided tests. Statistical analyses were conducted using R (version 3.4.1) (The R Project for Statistical Computing R) [13].

## Results

From 01.04 2004 through 31.12 2021, we performed surgical ablation AF in 632 patients, of whom 26 patients were excluded from the study due to incomplete data. Group I (non-diabetic patients), consisted of 484 patients, and Group II (DM type 2 patients), consisted of 122 patients. Patients with DM were older, had more arterial hypertension and incidence of CVA/TIA, and had a higher standard and logistic EuroSCORE I (*p* < 0.05 for all). There were no differences regarding sex, NIHA class, COPD, average systolic left ventricular injection fraction, and left ventricle and left atrial dimension between Groups I and II. (See Table 1). The mean follow-up duration was 39.0 ± 22.7 months. A 1-year follow-up was completed in 588 patients, a 3-year follow-up in 496 patients, and 5-year follow-up in 435 patients,

**Table 1:** Patients data

	Group I (Non-DM)	Group II (DM)	p Value
N	484	122	
Age	69 (62–75.3)	65 (57–72)	< 0.001
Male gender (n, %)	259 (53.5%)	71 (58.2%)	0.362
Elective surgery (n, %)	437 (90.3%)	104 (85.2%)	0.182
NIHA III-IV (n, %)	179 (37%)	44 (36.1%)	0.454
Previous cardiac surgery (n, %)	40 (8.3%)	5 (4.1%)	0.127
EF (%)	60 (51.5–60)	60 (53–60)	0.618
LVEDD (cm)	5 (4.5–5.5)	5.1 (4.6–5.6)	0.136
Left atrial volume (cm^3^)	113 (81–151)	123 (93–163)	0.054
Left ventricular dysfunction			
Moderate (n, %)	19 (3.9%)	8 (6.6%)	0.651
Severe (n, %)	6 (1.2%)	2 (1.6%)	
Paroxysmal AF	279 (57.6%)	68 (55.7%)	0.376
Persistent AF	149 (30.8%)	45 (36.9%)	0.76
Long-standing and permanent AF	56 (11.6%)	9 (7.4%)	0.231
Arterial hypertension (n, %)	288 (59.5%)	104 (85.2%)	< 0.001
COPD (n, %)	30 (6.2%)	14 (11.5%)	0.052
Dialysis (n, %)	1 (0.2%)	0 (0%)	1.000
Hyperlipidemia (n, %)	244 (50.4%)	92 (75.4%)	< 0.001
PVD (n, %)	20 (4.1%)	6 (4.9%)	0.614
CVA/TIA (n, %)	46 (9.5%)	19 (15.6%)	0.048
Pulmonary hypertension (n, %)	90 (18.6%)	19 (15.6%)	0.509
HA1C (%)	6.5 (5.9–7.3)	5.6 (5.3–5.8)	< 0.001
Standard EuroSCORE I	5 (4–7)	5 (3–6)	0.001
Logistic EuroSCORE I (%)	4.5 (2.7–8.1)	3.7 (2.1–5.8)	0.010

**Fig. 1:**
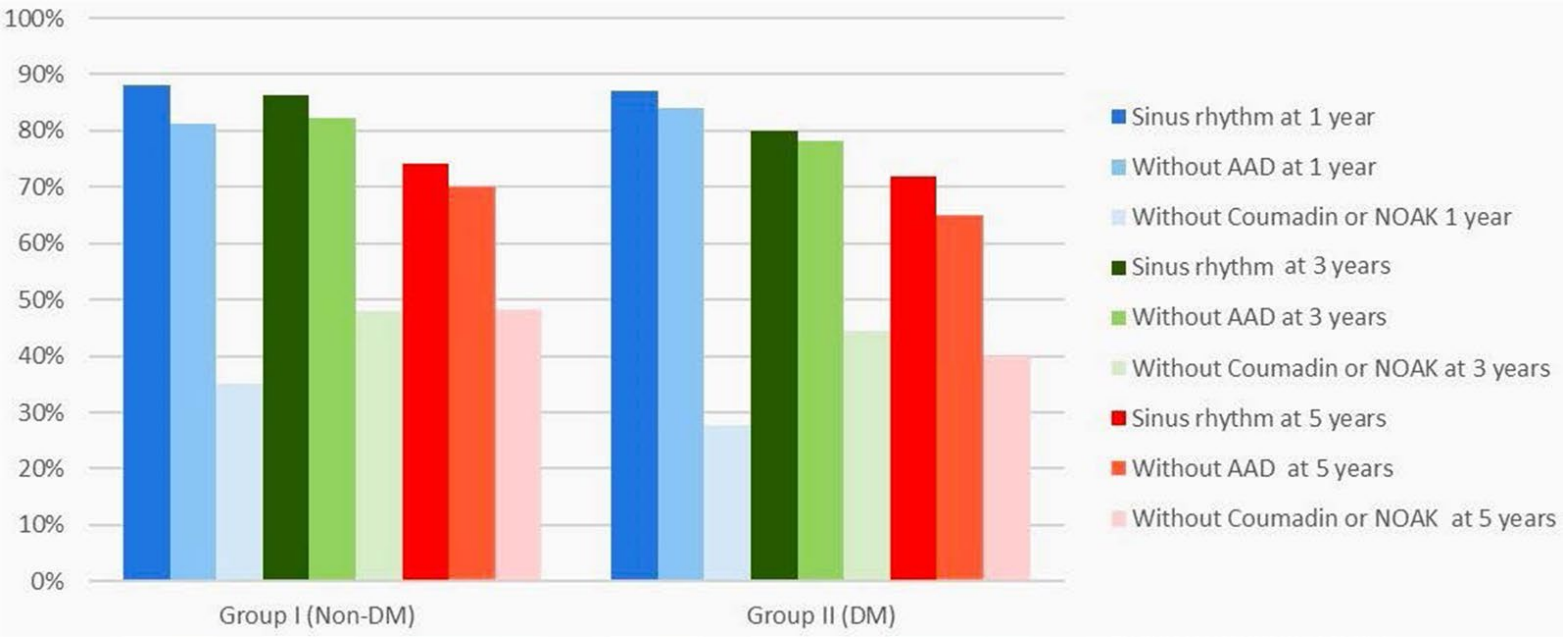
5-year rithm follow-up after surgical ablation

**Fig. 2:**
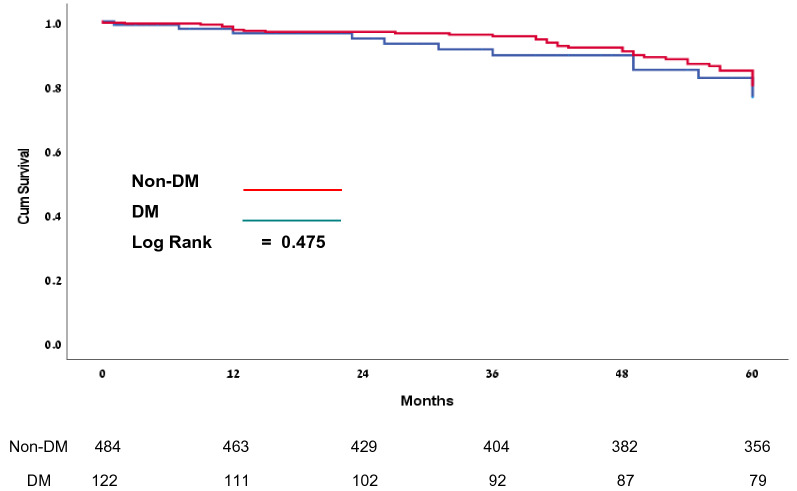
5-year freedom from recurrent AF

**Fig. 3:**
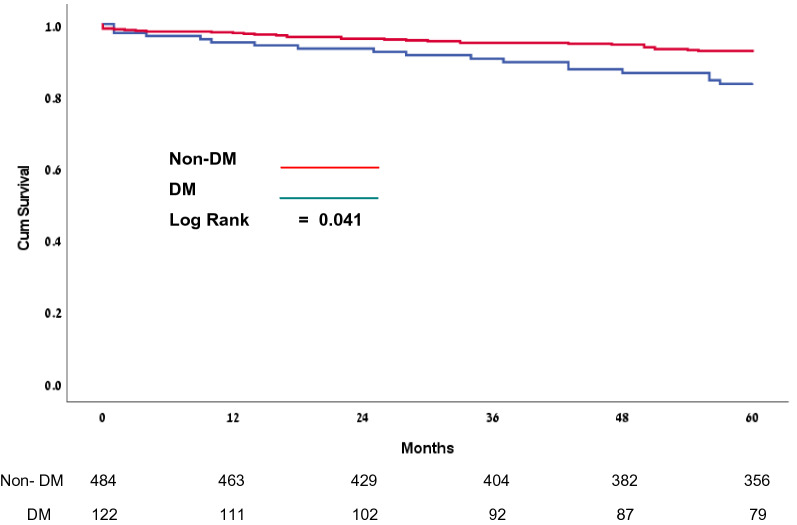
5-year survival after surgical ablation

Of this number of patients, 417 (84.7%) underwent isolated left atrial and 76 (12.5%) right and left atrial ablation with no differences between the groups (*p* = 0.067). DM type 2 patients (Group II) had a higher incidence of CABG (*p* < 0.001), also longer bypass of 130 ± 40 min vs. 122 ± 36 min (*p* = 0.028), and aortic cross-clamp time 104 ± 33 min vs. 95 ± 30 min (*p* = 0.003). Both groups had similar ventilation time, ICU, and hospital stay duration. Furthermore, 1- and 3-year mortality was similar between Group I and Group II, 2.5% vs. 4.9%, (*p* = 0.226) and 5.6% vs. 10.5% (*p* = 0.076) respectively. However, 5-year mortality was higher in Group II compared with Group I, 11.1% vs. 23.4% (*p* = 0.009) (See Table 2) Freedom from AF was similar between Group I and Group II after a 1-year follow-up, 414 (88.2%) vs. 101 (87.1%) (*p* = 0.511), after a 3-year follow-up, 360 (86.3%) vs. 84 (79.9%) (*p* = 0.290) and after a 5-year follow-up, 226 (74.1%) vs. 55 (71.5%) (*p* = 0.622) respectively (See Fig. 1). Kaplan—Meier analysis for freedom from atrial arrhythmias did not reveal a difference between the groups (*p* = 0.475) during follow-up (Fig. 2), however, Kaplan—Meier survival analysis showed a statistically significant difference in 5-year mortality between patients with and without DM type 2 (*p* = 0.041) (Fig. 3). By multiple logistic regression Cox analysis, we did not find factors that affected freedom from atrial arrhythmias, albeit we found that two factors affected patients’ 5-year mortality rates: diabetes mellitus and age (See Table 3).

**Table 2:** Perioperative data

	Group I (non-DM)	Group II (DM)	*p* Value
Included in the study (n)	484	122	
1-year follow-up (n)	472	116	
3-year follow-up (n)	404	92	
5-year follow-up (n)	356	79	
Ablation type			
Right + Left Atrial ablation (n,%)	53 (11%)	23 (18.8%)	0.067
Left Atrial ablation (n,%)	417 (86.2%)	97 (79.5%)	
Other ablation (n,%)	14 (2.9%)	2 (1.6%)	
Isolated MAZE procedure (n,%)	30 (6.2%)	8 (6.6%)	0.836
AV surgery (n,%)	90 (18.6%)	36 (29.5%)	0.012
MV surgery (n,%)	331(68.4%)	64 (52.5%)	0.001
TV surgery (n,%)	134 (27.7%)	23 (18.8%)	0.050
CABG (n,%)	79 (16.3%)	48 (39.3%)	< 0.001
Other (n,%)	60 (12.3%)	9 (7.4%)	0.026
Bypass time (min)	124 (102–150)	100 (80–121)	0.028
Cross-clamp time (min)	100 (80–121)	92 (75–114)	0.003
CVA/ TIA (n,%)	7 (1.4%)	7 (5.7%)	0.011
Low Cardiac Output (n,%)	6 (1.2%)	4 (3.3%)	0.121
Acute kidney injury (AKI) (n,%)	58 (13.7%)	22 (18%)	0.087
IABP (n,%)	2 (0.4%)	3 (2.5%)	0.058
Pacemaker (n,%)	15 (3.1%)	4 (3.3%)	1.000
ICU time (hours)	39 (19–72.8)	26 (20–60.8)	0.053
Ventilation time (hours)	12 (9–16.3)	11 (8–15)	0.064
Hospital length of stay (days)	11 (8–15)	9 (7–12)	0.305
1-year mortality (n,%)	12 (2.5%)	6 (4.9%)	0.226
3-year mortality (n,%)	24 (5.6%)	11 (10.5%)	0.076
5-year mortality (n,%)	34 (11.1%)	18 (23.4%)	0.009

**Table 3:** Risk factor for 5-Years AF recurrence and mortality

	B	Sig.	HR	95.0% CI for HR	
				lover	upper
Risk factor for 5-years AF recurrence					
Age	0.009	0.676	1.009	0.968	1.051
Gender	0.654	0.151	1.924	0.788	4.698
Diabetes mellitus	− 0.640	0.198	0.527	0.199	1.397
CABG	− 0.740	0.296	0.477	0.119	1.912
Cross-clamp time	0.011	0.180	1.011	0.995	1.029
Risk factor for 5-years mortality					
Age	− 0.487	< 0.001	0.002	1.265	2.345
Diabetes mellitus	0.018	< 0.001	1.018	1.008	1.029
Pulmonary hypertension	− 0.435	0.676	0.477	0.398	2.672

## Discussion

Our study is the first, that investigated the influence of DM type 2 on freedom from AF at 1- 3- and 5-year follow-up and 1- 3- and 5-year mortality after surgical AF ablation. DM type 2 is independently associated with new-onset AF, the coexistence of these two conditions contributes to poor outcomes [14], probably due to atrial remodeling following diffuse interstitial fibrosis initiated by the production of advanced glycation end-products [15]. The impact of DM type 2 on outcomes of surgical AF ablation is insufficiently described.

Surgical ablation has a high success rate and may improve cardiac function postoperatively. Gu et al. [16] reported, that the rate of sinus rhythm without anti-arrhythmic drugs at 12, 24, and 36 months was 78.3, 62.8 and 49.9%, respectively. Raissouni et al. [17] reported freedom from AF for 60% of patients after 1-year follow-up after concomitant cardiac surgery and radiofrequency AF ablation. Petersen et al*.* [18] reported 1-year survival 95.8% after the operation and the overall rate of freedom from AF ranged from 62 to 72%. Pecha et al. [19] found that surgical AF ablation provided freedom from AF rate of 63% at 12 months and 56.6% during long-term, 5.9 years follow-up, with significantly better results in patients with paroxysmal than in those with persistent AF (67.2% vs 51.8%, *p* = 0.03). Ad et al. [20] compared the outcomes of concomitant surgical ablation in the groups of patients with and without mitral valve procedures and found, that the proportion in sinus rhythm regardless of antiarrhythmic medication was similar in both groups at 1 year (88% vs 91%, *p* = 0.526), 3 years (83% vs 83%, *p* = 0.979), and 5 years after surgery (80% vs 72%, *p* = 0.303). These results are very similar to our results: 88.2–87.1% after a 1-year follow-up, 86.3–79.9% after a 3-year follow-up, and 74.1–71.5% after a 5-year follow-up, respectively. Surgical ablation is a very effective treatment for AF, because all lesions, both using bipolar RF clamps or cryoablation probes are fully transmural, and start or end in electrically inactive tissue [21].

It should be noted that none of the above-mentioned studies report DM type 2 as a risk factor for AF recurrence after surgical ablation. In contrast, DM type 2 has an effect on the outcome of patients who underwent catheter ablation. Wang et al*.* [22], reported, that arrhythmia recurrence after catheter ablation was significantly higher in the DM type 2 group compared to the no-DM group after adjustment for baseline differences (HR 2.24; CI_95%_ 1.42–3.55; *p* = 0.001). There was a nonsignificant trend toward higher AF recurrence in patients with worse glycemic control. Creta et al. [23] investigated cryoballoon ablation and reported, that arrhythmia relapses at 12 months after AF ablation occurred more frequently in the DM type 2 group (32.0% vs 25.3%, p = 0.031). These results were confirmed in a propensity-matched analysis, and DM type 2 was also an independent predictor of AF recurrence on the multivariate analysis (HR 1.39; CI _95%_1.07 to 1.88; *p* = 0.016). Kim et al. [24] studied 2,352 patients with AF, undergoing first-time radiofrequency ablation in a single institution, and found, that diabetes was associated with increased risk of recurrent AF after 5 years (HR = 1.34; *p* = 0.015).

Furthermore, we found that 1- and 3-year mortality after concomitant surgical ablation was similar between DM type 2 and non-DM groups, but 5-year mortality was higher in the DM type 2 group. These data are comparable to other studies investigating the impact of diabetes on mortality after other types of cardiac surgery. Koshizaka et al. [25] evaluated 3,014 patients after CABG in PREVENT IV study. At 5 years, rates of death, myocardial infarction, or revascularization were higher among DM type 2 patients compared with those non-DM patients (adjusted hazard ratio 1.57; 95% CI 1.26–1.96; P < 0.001). Kogan et al. [26] performed an observational cohort study in 2766 patients after the first time isolated CABG, who were divided into two groups: Group I (1553 non-DM patients), and Group II (1213 DM type 2 patients). 5- and 10- years mortality was higher in DM patients compared with non-DM patients (15.3% vs. 9.3%, p < 0.001, and 47.3% vs. 29.6% p < 0.001). Multivariable analysis showed that DM type 2 increased the mortality risk by twofold. Ram et al. [27] studied 1053 patients, after isolated aortic valve surgery. Early mortality was higher in diabetic compared with non-diabetic patients. Long-term (5- and 10-year) mortality was significantly higher in the DM type 2 patients, compared to the patients without diabetes: 19.4% vs. 12.9% (p = 0.007) and 30.3% vs. 23.5% (p = 0.020) respectively. Veiga-Oliveira et al. [28] described a single-center experience with 130 patients who underwent complex triple-valve surgery. Most of the patients were female (72.3%), mean age of 64.4 years; 61.1% were in New York Heart Association class III/IV. The survival at 5 and 10 years was 60% and 43%, respectively. Multivariable analyses show, that DM was a risk factor for long-term mortality.

## Limitations

There are a few limitations in our study. First, while it is retrospective in design. Secondly, our study was conducted in a single-center cardiac surgery department. Other hospitals may have different experiences. Third, within the DM group of patients, we do not compare the outcome of insulin-treated vs. non-insulin-treated patients. And fourth, the authors do not collect information regarding the adequacy of glycemic control before surgery.

## Conclusions

Surgical ablation has a high success rate. First, our principal finding was that the freedom from recurrent atrial arrhythmia at 1- 3- and 5- years was similar in the DM type 2 group compared to the non-DM group. Second, we found that among patients undergoing AF surgical ablation, DM type 2 and non-DM patients had similar 1- and 3-year mortality, albeit diabetes was associated with increased 5-year mortality.

## Data Availability

Data collected from a departmental database. The datasets used and analysed during the current study are available from the corresponding author on reasonable request.

## Abbreviations

AF: Atrial fibrillation
AVR: Aortic valve replacement
DM: Diabetes mellitus
CVA: Cerebrovascular accident
TIA: Transient ischemic attack
RF: Radiofrequency
ICU: Intensive Care Unit
AT: Atrial tachyarrithmia
SD: Standard deviation
CABG: Coronary artery bypass grafting
