# Correction: Surgical ablation for atrial fibrillation: impact of Diabetes Mellitus type 2

**DOI:** 10.1186/s12933-023-01851-2

**Published:** 2023-05-18

**Authors:** Alexander Kogan, Avishay Grupper, Avi Sabbag, Eilon Ram, Tamer Jamal, Eyal Nof, Enrique Z. Fisman, Shany Levin, Roy Beinart, Jonathan Frogel, Ehud Raanani, Leonid Sternik

**Affiliations:** 1 Department of Cardiac Surgery, Sheba Medical Center at Tel Hashomer, 52621 Ramat Gan, Israel; 2 Division of Cardiology, Sheba Medical Center at Tel Hashomer, 52621 Ramat Gan, Israel; 3 Department of Anesthesiology, Sheba Medical Center at Tel Hashomer, 52621 Ramat Gan, Israel; 4 Sackler School of Medicine, Tel Aviv University, Tel Aviv, Israel

**Correction: Cardiovascular Diabetology (2023) 22:77**

10.1186/s12933-023-01810-x

Following publication of the original article [1], the conflict of interest statement has been updated with this correction.

## Conflict of Interest

Enrique Fisman declares that he is Editor-in-Chief of *Cardiovascular Diabetology*. This article was assigned to another Editor to assume responsibility for overseeing peer review. This submission was subject to the exact same review process as any other manuscript submitted to the journal.
